# Use of Enamel Matrix Derivative in Minimally Invasive/Flapless Approaches: A Systematic Review with Meta-Analysis

**DOI:** 10.3290/j.ohpd.b3125655

**Published:** 2022-06-13

**Authors:** Nathan E. Estrin, Vittorio Moraschini, Yufeng Zhang, Richard J. Miron

**Affiliations:** a Periodontist, Lake Erie College of Osteopathic Medicine School of Dental Medicine, Bradenton, FL, USA. Study concept and design, performed the literature search, interpretation of the data, drafted and revised the manuscript critically for important intellectual content, approved the final submitted version, accountable for all aspects of the study design and its content.; b Professor and Periodontist, Department of Periodontology, Dental Research Division, School of Dentistry, Veiga de Almeida University, Rio de Janeiro, Brazil. Study concept and design, performed the literature search, interpretation of the data, drafted and revised the manuscript critically for important intellectual content, approved the final submitted version, accountable for all aspects of the study design and its content.; c Professor and Oral Surgeon, Department of Oral Implantology, Wuhan University, China. Study concept and design, drafted and revised the manuscript critically for important intellectual content, approved the final submitted version, accountable for all aspects of the study design and its content.; d Research Associate, Department of Periodontology, University of Bern, Bern, Switzerland. Study concept and design, performed the literature search, interpretation of the data, drafted and revised the manuscript critically for important intellectual content, approved the final submitted version, accountable for all aspects of the study design and its content.

**Keywords:** EMD, enamel matrix derivative, enamel matrix proteins, intrabony defect, minimally invasive surgery

## Abstract

**Purpose:**

The aim of the present systematic review with meta-analysis was to investigate the clinical effectiveness of EMD (enamel matrix derivative) using a minimally invasive surgical technique (MIST) or flapless approach for the treatment of severe periodontal probing depths.

**Materials and Methods:**

A systematic review of the literature including searches in PubMed/Medline, Cochrane Library, Google Scholar, and Grey Literature databases as well as manual searches was performed on September 1st, 2021. Studies utilising EMD in a non-surgical or minimally invasive approach were included. The eligibility criteria comprised randomised controlled trials (RCTs) comparing minimally-invasive/flapless approaches with/without EMD for the treatment of probing depths >5 mm.

**Results:**

From 1525 initial articles, 7 RCTs were included and 12 case series discussed. Three studies investigated a MIST approach, whereas 3 studies utilised a flapless approach. One study compared EMD with either a MIST or a flapless approach. The RCTs included ranged from 19–49 patients with at least 6 months of follow-up. While 5 of the studies included smokers, patients smoking >20 cigarettes/day were excluded from the study. The meta-analysis revealed that EMD with MIST improved recession coverage (REC) and bone fill (BF) when compared to MIST without EMD. However, no difference in CAL or PD was observed between MIST + EMD vs MIST without EMD. No statistically significant advantage was found for employing the EMD via the flapless approach.

**Conclusions:**

Implementing EMD in MIST procedures displayed statistically significant improvement in REC and BF when compared to MIST alone. These findings suggest that MIST in combination with EMD led to improved clinical outcomes while EMD employed in nonsurgical flapless therapy yielded no clinical benefits when compared to nonsurgical therapy alone without EMD. More research is needed to substantiate these findings.

Periodontal disease is one of the most prevalent chronic diseases known to humans. It begins as a superficial inflammatory response of the gingiva (gingivitis) and later progresses to attachment loss with subsequent destruction of the tooth-supporting structures (periodontitis).^2,20,23,31,34^ The goals of periodontal therapy include prevention of further progression of the disease, and if possible, to regenerate previously lost periodontal tissues.^4,21,30,39^ Results investigating the distribution of the disease from a national survey conducted in the USA found that over 47% of the adult population was affected, with 38.5% of the population having either moderate or severe cases (stage III or stage IV).^6^ This finding is most alarming, as the disease is characterised by an exponentially more difficult resolution and regeneration once advanced progression and loss of the periodontium has taken place.

Growth factors have commonly been utilised to further assist in the treatment of intrabony defects by providing signaling molecules necessary for the regeneration of the periodontium. One well-documented strategy, for which over 20 years of data (and over 1000 studies) have now accumulated, is the use of enamel matrix derivative (EMD; Emdogain, Straumann; Basel, Switzerland) as an adjunct to periodontal therapy.^26^ Over 25 years ago, a team of researchers observed that enamel matrix proteins (EMPs), which until then were considered an enamel-specific protein, were deposited onto the surface of developing tooth roots prior to cementum formation.^14^ This observation led to the hypothesis that EMPs may play an integral role in the future differentiation of periodontal tissues prior to cementum formation.^14^ This hypothesis was further investigated in a number of animal and clinical histological studies, demonstrating that EMPs were secreted by Hertwig’s epithelial root sheath and able to promote periodontal regeneration.^8,9,14,15,18,19,42^ The purified fraction derived from the enamel layer of developing porcine teeth was given the working name enamel matrix derivative (EMD) and has been the basis of numerous publications investigating its use in periodontal regeneration.^26^

To date, the majority of clinical studies using EMD for the management of periodontal disease has been accompanied by surgical approaches.^26^ While a growing number of studies are accumulating using EMD as an adjunct to non-surgical (flapless) or minimally invasive surgical techniques (MIST), results remain inconclusive. Furthermore, while a variety of studies have shown no additional benefit using EMD in non-surgical/minimally-invasive therapy,^10,13,28,35,41^ others have demonstrated positive outcomes.^1,11,16,17,24,40^

The aim of the present systematic review with meta-analysis was therefore to gather and evaluate the current evidence regarding the treatment of periodontal pockets using flapless/minimally invasive approaches both with and without EMD. The null hypothesis was that there are no beneficial effects of additionally using EMD on the clinical outcomes of either MIST or flapless approaches. Furthermore, all case series were documented and guidelines/recommendations from the authors regarding inclusion criteria and surgical considerations are discussed to better explore treatment outcomes using EMD in non-surgical/minimally invasive therapy.

## Materials and Methods

### Protocol

This systematic review followed the recommendations of the PRISMA guidelines.^27^ The protocol for this systematic review was based on PRISMA-P.^36^ There were no deviations from the initial protocol.

### Focused Question

What is the effectiveness of EMD for the treatment of periodontal pockets using a minimally-invasive or flapless approach?

### Eligibility Criteria and Study Selection Process

The inclusion criteria were based on the PICOS strategy.^33^ The search-and-screening process was conducted by two independent reviewing authors (NEE and RJM) commencing with the analysis of titles and abstracts. Next, full papers were selected for careful reading and matched with the eligibility criteria for future data extraction. Disagreements between the reviewing authors were resolved through careful discussion. Only studies meeting the following criteria were included:

- Population: Systemically healthy humans with active periodontal pockets greater than 5 mm;
- Intervention: EMD used adjunctively to either a MIST or flapless approach;
- Comparison: EMD in MIST/flapless approach vs MIST or nonsurgical periodontal therapy without EMD;
- Outcomes: The main outcome variable was the change in pocket depth (PD), and secondary outcome variables were clinical attachment level (CAL) and bone fill (BF) if reported (the flapless approaches did not include this);
- Study design: RCTs and case series.

### Search Strategy

PubMed/MEDLINE, the Cochrane Central Register of Controlled Trials, Scopus, Embase, and Lilacs were used to search for articles that were published before September 1st, 2021 without other restrictions regarding date or language. A search of the gray literature using the Literature Report and OpenGrey databases was also conducted. Finally, the study reference lists were evaluated (cross-referenced) to identify other studies for potential inclusion.

### Data Synthesis

The study data were extracted by NEE and RJM and systematically reviewed by VM. The following data, when available, were extracted from the included studies: authors, study design, follow-up, number of subjects, age range, gender, number of smokers, surgical technique, mean difference (MD) in PD, CAL, and BF.

### Risk of Bias within Studies

The two reviewing authors (VM and RM) analysed the risk of bias. The RoB 2 (a revised Cochrane risk-of-bias tool for randomised trials)^37^ was used to analyse the risk of bias in RCTs. Each study was analysed in relation to five domains: risk of bias arising from the randomisation process, risk of bias due to deviations from the intended interventions, missing outcome data, risk of bias in the measurement of the outcome, and risk of bias in the selection of the reported research. Studies were classified as having low risk, some concerns, or high risks of bias for each domain. The overall risk of biased judgment involved the following criteria: low risk, when the five areas of the study were judged as low risk; some concerns, when the study is judged as raising some concerns in at least one area; and high risk, when the study is judged to be at high risk in at least one domain or when the study is judged to have some concerns for multiple domains in a way that substantially lowers confidence in the results.

### Statistical Analysis

The continuous variables (CAL, PD, REC, and BF) of the included studies were categorised in subgroups and analysed in a meta-analysis using Review Manager software (version 5.2.8; Copenhagen, Denmark, 2014).

The estimates of the intervention effects (i.e. the MD) were expressed as percentages or millimeters with 95% confidence intervals (CI). The inverse variance method was used for the random effect or fixed-effect models, depending on the heterogeneity between the studies. Chi^2^ tests evaluated the heterogeneity, considering it to be low for values ≤ 25%, moderate for values > 25 ≤ 50%, and high for values > 50%.^5^ For cases of low or medium heterogeneity, the random effect model evaluated the variance components in the presence of heterogeneity (p < 0.10) rather than the fixed-effect model. The statistical significance level of the meta-analysis effect was set at p < 0.05.

## Results

### Literature Search

The process of the search, selection, and the reasons for excluding potential studies are shown in supplemental Fig 1. Seventeen studies on periodontitis treated with EMD adjunctive to a flapless or minimally invasive approach published between 2005 and 2021 were discussed, with 7 studies meeting the eligibility criteria and were included in the meta-analysis. Of the 7 RCTs, three studies investigated the use of a minimally invasive surgical technique (MIST) with/without EMD, whereas 3 studies investigated EMD used with a flapless approach compared to nonsurgical therapy without EMD. One study compared using a MIST approach with EMD vs a flapless approach with EMD. Five of seven studies included smokers.

**Fig 1:**
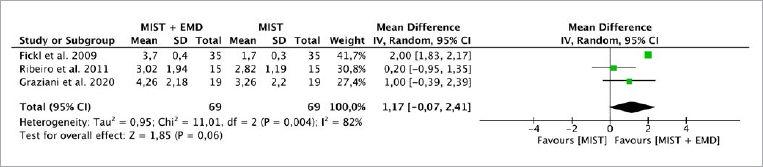
Forest plot for the event “clinical attachment level” (CAL) (reported in mm) for intrabony defects treated with MIST vs MIST + EMD.

### Meta-Analysis

#### MIST + EMD vs MIST without EMD

**Fig 2:**
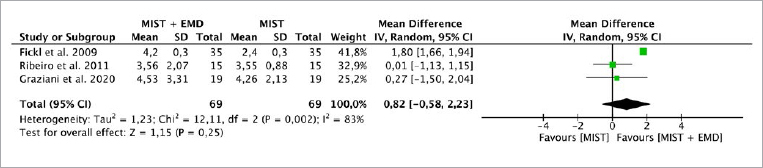
Forest plot for the event “reduction in probing depth” (PD) (reported in mm) for intrabony defects treated with MIST vs MIST + EMD.

**Fig 3:**
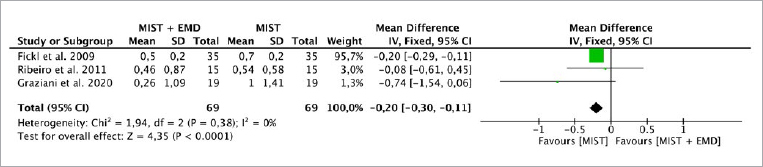
Forest plot for the event “reduction in recession coverage” (REC) (reported in mm) for intrabony defects treated with MIST vs MIST + EMD.

Three studies^7,12,29^ evaluated the CAL, PD and REC parameters, while two studies^7,29^ evaluated the BF parameter. The random-effects model was used to evaluate CAL and PD due to the high heterogeneity between the studies (p = 0.004, I^2^ = 82%; p = 0.002, I^2^ = 83, respectively). There was no statistically significant difference for CAL between the MIST alone (without EMD) vs MIST + EMD groups (p = 0.06), with a MD of 1.17 (95% CI: -0.07 to 2.41) and PD (p = 0.25), with an MD of 0.82 (95% CI: -0.58 to 2.23) (Figs 1 and 2). The fixed-effect model was used to evaluate REC due the absence of heterogeneity between studies (p = 0.38; I^2^ = 0%). There was a statistically significant difference (p < 0.0001), with an MD of -0.20 (95% CI: -0.30 to -0.11) in favor of MIST plus EMD for REC when compared to MIST alone without EMD (Fig 3).

**Fig 4:**
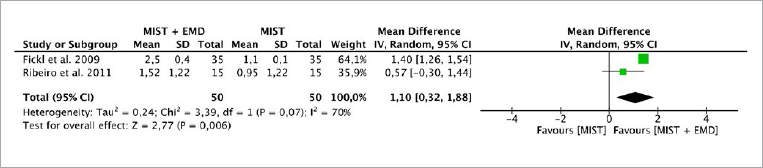
Forest plot for the event “reduction in bone fill” (BF) (reported in mm) for intrabony defects treated with MIST vs MIST + EMD.

The random-effects model was used to investigate bone fill due to the high heterogeneity between the studies (p = 0.07, I^2^ = 70%). There was a statistically significant difference in favor of EMD in combination with MIST (p = 0.006), with an MD of 1.10 (95% CI: 0.32 to 1.88) when compared to MIST alone without EMD (Fig 4).

#### SRP + EMD vs SRP without EMD

**Fig 5:**
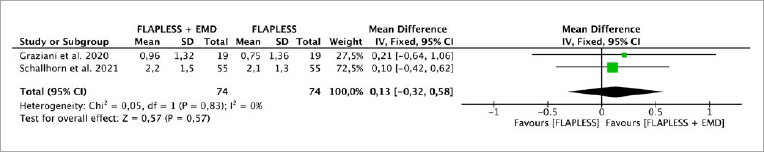
Forest plot for the event “clinical attachment level” (CAL) (reported in mm) for intrabony defects treated with a flapless technique with/without EMD.

**Fig 6:**
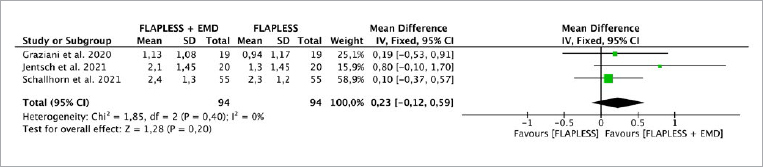
Forest plot for the event “reduction in probing depth” (PD) (reported in mm) for intrabony defects treated with a flapless technique with/without EMD.

Two studies^12,32^ evaluated the CAL parameter, while three studies^12,22,32^ evaluated PPD. The fixed-effect model was used to evaluate CAL and PPD due the absence of heterogeneity between the studies (p = 0.83, I^2^ = 0% and p = 0.40, I^2^ = 0%, respectively). There was no statistically significant difference for CAL (p = 0.57), with an MD of 0.13 (95% CI: -0.32 to 0.58) and PPD (p = 0.20), with an MD of 0.23 (95% CI: -0.12 to 0.59), when SRP plus EMD was compared with SRP alone without EMD (Figs 5 and 6).

### Risk of Bias within Studies

Two studies^7,29^ present a biased judgment, classified as “some concern”. These studies showed the possibility of bias in the randomisation process. All other studies were classified as “low risk of bias.” The ROB 2 analysis is shown in Supplemental Table 1.

**Table 1:** Main characteristics of the included case series studies

Authors (year)	Study design Follow-up	Inclusion criteria Access technique	No. of participants Gender Mean age	Groups (# of Sites)	Smokers (No, Yes)	Conclusions
Minimally invasive						
Harrel et al (2005, 2010)	Case series 6 years	PPD > 6 mm MIS	13 ♂8 / ♀5	142	No	MIST with EMP yielded significant improvements in periodontal parameters and periodontal conditions remained stable since the 6 year follow-up study.
Cortellini and Tonetti (2007)	Case cohort 1 year	PPD > 5 mm MIST	13 ♂4 / ♀9 43.1 ± 9.8	13	No	The technique of MIST in combination with EMD alone presented significant improvement in periodontal parameters in this limited patient pool.
Cortellini et al (2007, 2009)	Case cohort 1 year	PPD > 5 mm MIST	40 ♂14 / ♀26 48.3 ± 9.8	40	Yes (<10 cigarettes)	MIST in conjunction with EMD presented significant improvement in periodontal parameters.
Cortellini et al (2008)	Case cohort 1 year	PPD > 5 mm MIST	20 ♂6 / ♀14 49.7 ± 8.3	44	Yes (<10 cigarettes)	MIST in combination with EMD is an acceptable treatment to successfully regenerate multiple deep intrabony defects.
Miliauskaite et al (2008)	Case cohort 3 years	PPD > 6 mm PPT	25 ♂14 / ♀11 Age range: 28–68	60	No	PPT combined with EMD resulted in significant improvement of periodontal parameters.
Ribeiro et al (2010)	Case series 6 months	PPD > 5 mm MIST	12 ♂5 / ♀7 47.4 ± 7.0	12	No	MIST combined with EMD promotes satisfactory clinical outcomes.
Harrel et al (2014, 2016, 2017)	Prospective cohort 36–58 months	PPD > 5 mm V-MIS	14 ♂6 / ♀12 54.6 ± 11.6	22	No	V-MIS combined with emdogain provided improvement in the parameters PPD and CAL with no post-surgical recession.
Flapless						
Aimetti et al (2021)	Prospective case series 2 years	CAL > 6 mm Flapless	11 ♂6 / ♀5 44.6± 9.4	11	No	All defects treated with EMD in combination with the flapless approach presented with favorable outcomes.

RCT: randomised clinical trial; NR: not reported; C: control group; T: test group; ♂male; ♀female; PPD: pocket probing depth; CAL: clinical attachment level; MIS: minimally invasive surgery; MIST: minimally invasive surgical technique; V-MIS: videoscope assisted minimally invasive surgery; FMPS: full-mouth plaque score.

## Discussion

The present systematic review with meta-analysis investigated the use of EMD in either MIST or flapless procedures. Overall it was found that the MIST protocol led to significant improvements in REC and BF (Figs 3 and 4) when EMD was utilised, whereas no differences were reported when comparing a flapless procedure (SRP) with and without EMD. While a number of case reports have now utilised EMD in combination with either a flapless or MIST protocol (Table 1), only 7 studies investigated the results in RCTs (Table 2). Interestingly, many of the case series generally found improvements in CAL ranging from 3 to 4.8 mm and improvement in PD reduction ranging from 3.2 to 5.2 mm (Table 1). Furthermore, little change in recession coverage was observed following minimally invasive regenerative therapy, while statistically significant improvements in CAL and PD were noted.

**Table 2:** Main characteristics of the included randomised clinical studies on intrabony defects

Authors (year)	Study design Follow-up	Inclusion criteria Access technique	No. of participants Gender Mean age	Groups	Smokers (No, Yes)	Conclusions
Minimally Invasive						
Fickl et al (2009)	RCT (split-mouth) 12 months	PPD > 6mm Microsurgical access flap	19 ♂6 / ♀13 46.1	C: 35, MIST T: 35, MIST + EMD	Yes (<10 cigarettes)	The EMD group displayed improved clinical parameters including a higher PPD reduction, CAL gain, and radiographic bone fill.
Ribeiro et al (2011)	RCT (parallel) 6 months	PPD > 5 mm MIST	30 ♂11 / ♀19 47.1	C: 15, MIST T: 15, MIST + EMD	No	No statistically significant differences were observed between the test and control groups.
Graziani et al (2020)	RCT (parallel) 6 months	PPD > 5 mm MIS	38 ♂18 / ♀20 54.92	C: 19, MIS T: 19, MIS + EMD	Yes (<20 cigarettes)	CRP was higher in the EMD group 24 h after surgery. The EMD group also displayed less recession than the control group.
Flapless						
Graziani et al (2019)	RCT (parallel) 3 months	CAL > 3mm Flapless	38 ♂18 / ♀20 50.5	C: 19, SRP T: 19, SRP + EMD	Yes (<20 cigarettes)	The EMD group resulted in lower fibrinolysis and better periodontal healing.
Jentsch et al (2021)	RCT (split-mouth) 12 months	PPD > 5 mm and < 8mm Flapless	44 ♂21 / ♀23 Age range: 31-74	C: 20, reinstrumentation T: 20, reintstrumentation + EMD	Yes (<10 cigarettes)	The EMD group displayed a significant increase in PPD reduction compared to the control.
Schallhorn et al (2021)	RCT (split-mouth) 12 months	PPD > 5 mm and < 8mm Flapless	55 ♂51% / ♀49% 55.2	C: 55, SRP T: 55, SRP + EMD	Yes (<10 cigarettes)	The test group displayed fewer sites with BOP and a higher number of healthy PPDs compared to the control.
Both groups						
Aimetti et al 2017	RCT (parallel) 24 months	PPD > 6 mm MIST (C)/Flapless (T)	30 ♂18 / ♀12 43.25	C: 15, MIST + EMD T: 15, Flapless + EMD	No	Both groups yielded similar results in PD reduction and CAL gain. Therefore the flapless procedure can be utilised and achieve comparable results to the MIST technique.

Authors (year)	Mean difference in CAL between baseline and final follow-up (mm)	Mean difference in PD between baseline and final follow-up (mm)	Mean difference in REC between baseline and final follow-up (mm)	Radiographic bone fill (mm)	Converted pockets (<5 mm) (%)	Plaque score (Baseline/follow-up)		Bleeding score (Baseline/follow-up)	
Minimally invasive									
Fickl et al (2009)	1.7 ± 0.3 (C) 3.7 ± 0.4 (T)	2.4 ± 0.3 (C) 4.2 ± 0.3 (T)	0.7 ± 0.2 (C) 0.5 ± 0.2 (T)	1.1 ± 0.1 (C) 2.5 ± 0.4 (T)	NR	30.53 + 3.89 (API)	24.68 + 5.88 (API)	29% (C) 26% (T)	37% (C) 0% (T)
Ribeiro et al (2011)	2.82 ± 1.19 (C) 3.02 ± 1.94 (T)	3.55 ± 0.88 (C) 3.56 ± 2.07 (T)	0.54 ± 0.58 (C) 0.46 ± 0.87 (T)	0.95 ± 1.22 (C) 1.52 ± 1.22 (T)	NR	15.79 + 6.04 (C) 16.91 + 3.22 (T) (FMPS)	10.71 + 4.70 (C) 13.71 + 4.13 (T) (FMPS)	9.33 + 5.39 (C) 11.99 + 4.56 (T)	6.15 + 3.63 (C) 7.65 + 3.16 (T)
Graziani et al (2020)	3.26 ± 2.20 (C) 4.26 ± 2.18 (T)	4.26 ± 2.13 (C) 4.53 ± 2.31 (T)	1.00 ± 1.41 (C) 0.26 ± 1.09 (T)	NR	26.31 (C) 57.89 (T)	NR		NR	
Flapless									
Graziani et al (2019)	0.75 ± 1.36 (C) 0.96 ± 1.32 (T)	.94 ± 1.17 (C) 1.13 ± 1.08 (T)	NR	NR	10.61 + 4.57 (C) 11.21 + 3.93 (T)	60.31 + 22.73 (C) 65.76 + 20.98 (T) (FMPS)	14.66 + 12.65 (C) 10.85 + 13.76 (T) (FMPS)	60.36 + 22.64 (C) 55.71 + 17.92 (T)	20.43+ 20.48 (C) 13.78 + 14.53 (T)
Jentsch et al (2021)	NR	1.3 ± 1.45 (C) 2.1 ± 1.45 (T)	NR	NR	45 (C) 80 (T)	NR		100 (C) 100 (T)	22.5 (C) 5.0 (T)
Schallhorn et al (2021)	2.1 ± 1.3 (C) 2.2 ± 1.5 (T)	2.3 ± 1.2 (C) 2.4 ± 1.3 (T)	-0.2 ± 1.0 (C) -0.3 ± 0.9 (T)	NR	65.9 (C) 79.8 (T)	55.1 (C) 54.3 (T) (FMPS)	30.2 30.2 (FMPS)	56.1 (C) 54.6 (T)	23.3 (C) 17.8 (T)
Both groups									
Aimetti et al 2017	3.6 ± 0.9 (C) 3.2 ± 1.1 (T)	3.7 ± .6 (C) 3.6 ± 1.0 (T)	-0.1 ± 0.5 (C) -0.4 ± 0.7 (T)	3.8 ± 1.3 (C) 2.6 ± 1.6 (T)	53.33 (C) 66.67 (T)	10.7 + 2.4 (C) 11.9 + 2.0 (T) (FMPS)	10.9 + 1.9 (C) 11.5 + 1.6 (T) (FMPS)	8.3 + 2.1 (C) 8.8 + 2.9 (T)	8.7 + 1.8 (C) 9.5 + 1.6 (T)

RCT: randomised clinical trial; NR: not reported; C: control group; T: test group: ♂male; ♀female; PPD: pocket probing depth; CAL: clinical attachment level; MIS: minimally invasive surgery; MIST: minimally invasive surgical technique; SRP: scaling and root planing; FMPS: full-mouth plaque score; API: approximal plaque index.

Several advantages were also reported when EMD was combined with either MIST or flapless procedures, as noted in various studies included in this systematic review. For instance, while Schallhorn et al^32^ did not find statistically significant changes in PPD and CAL between using a MIST vs MIST + EMD, the EMD group displayed a greater amount of overall probing depths converting to sites no longer requiring surgical treatment (pockets < 5 mm), with 79.8% of test sites compared to 65.9% of control sites. Those authors also showed a greater decrease in number of sites exhibiting BOP in the test group compared to the control group. This is consistent with Graziani et al,^11^ who also showed a greater number of overall converted sites displaying probing depths < 5 mm and number of BOP in the test group compared to the control group (11 sites vs 5 sites). Fickl et al^7^ also reported a greater reduction in BOP in the test group compared to the control; after 12 months, 0% BOP was observed in the EMD group, while 37% remained in the control group. Additionally, Jentsche et al^22^ showed a greater decrease in BOP in the test group (5%) compared to the control (22.5%).

In 2020, Graziani et al^12^ investigated the amount of C-reactive protein (CRP) in each group at 24 h post-surgery to determine the effect of EMD on the inflammatory response. Lower values of CRP and fibrinogen were observed at 24 h in the EMD group than in the control group, suggesting a reduced inflammatory response when EMD was utilised.^12^ Furthermore, in the same study, utilising a flapless approach plus EMD (in which no overall statistically significant difference in PPD reduction was found), sites with PPD ≥ 6 mm exhibited statistically significant improvement in the EMD group when compared to the flapless approach alone (3.25 mm vs 2.11 mm).^11^

One of the main observations was the variability between the RCTs with respect to the use of EMD. For instance, Ribeiro et al^29^ found that additional use of EMD led to a CAL gain of only 0.2 mm (non-significant), whereas Fickl et al^7^ demonstrated a statistically significant improvement in CAL of 2 mm when EMD was combined with MIST. Therefore, it appears that this large variability may be attributed to differences in surgical technique or other factors; more data is needed to better understand factors related to these observed outcomes.

One plausible explanation for differences in regenerative outcomes while using EMD may be attributed to the adsorption kinetics of EMD to the root surface. Previously, in a study titled “Enamel matrix protein adsorption to root surfaces in the presence or absence of human blood”,^25^ it was found that blood in general had a negative impact on the adsorption of EMD to root surfaces. It was revealed that plasma proteins from blood samples altered the ability of EMD to adsorb to root surfaces on human teeth.^25^ In contrast, root surfaces coated with EMD lacking blood demonstrated a consistent, even layer of EMD adsorption to the root surface, which was further shown to improve PDL cell attachment and proliferation when compared to samples containing blood.^25^ Thus, it appears that thorough removal of blood remnants and bleeding during the clinical application of EMD onto the root surface is a significant factor and one that is rarely discussed during the surgical application of EMD. Importantly, without proper adsorption of EMD onto the root surface, no clinical benefits of EMD should be expected. This may partly explain why better results were observed in the meta-analysis when EMD was utilised in MIST as opposed to a flapless procedure, where more difficulty in removing all blood smears on the root surface may have been encountered. Further research specifically investigating these factors is needed, including histological data to determine which surgical techniques are best to adsorb enamel matrix proteins on the root surface.

A second factor influencing the outcomes may also be the clinician’s ability to completely remove calculus when scaling and root planing in the flapless approach vs MIST. In a classic study by Caffesse et al,^3^ it was revealed that scaling in pockets from 4–6 mm and greater than 6 mm both demonstrated a statistically significantly higher percentage calculus-free area when flaps were raised compared to a flapless approach. Thus, the extent of residual calculus was directly related to pocket depth and was greatest at the CEJ or in association with grooves, fossae or furcations.^3^ Thus, depending on tooth morphology, a MIST approach may be preferable to the flapless method. Future research is needed for clinical guidelines that better address when to select a flapless vs MIST approach.

Furthermore, 5 out of 7 studies included smokers. Given that smoking negatively affects periodontal regeneration, the improvement in clinical parameters may be limited in this study. More RCTs with control of local and systemic factors are needed to accurately assess the regenerative potential of EMD in these treatment approaches in both smokers and non-smokers.

While the MIST + EMD group showed statistically significant improvement when compared to the flapless + EMD group, it is hard to make a true comparison, due to potential differences in the subjects and defect types among treatment groups. Studies evaluating MIST + EMD treated patients with intrabony defects, whereas studies evaluating a flapless approach treated all patients with periodontal pockets, and having intrabony defects was not mandatory for inclusion in these studies. According to Tonetti et al,^38^ defect characteristics play a statistically significant role in the amount of potential regeneration achieved, with deeper vertical defects achieving greater tissue gain. Therefore, the MIST group may have had an advantage resulting in more improvement in periodontal parameters when compared to flapless group for these reasons. However, Aimetti et al^1^ compared the flapless and MIST approaches when having an intrabony defect was an inclusion criterion for both groups. They reported no statistically significant difference between the two approaches. More studies are therefore needed that evaluate the same type of bony defects in order to more accurately compare MIST vs flapless approaches in conjunction with EMD and provide adequate clinical guidelines.

Another limitation of this study is that there were differences in the phasing of periodontal therapy. For instance, not all studies had patients undergo initial non-surgical therapy prior to the intervention evaluated. The inclusion criteria of two studies stated that no periodontal treatment was conducted 6 months prior to treatment,^11,32^ while four other studies reported that the patients had undergone nonsurgical therapy prior to beginning the study.^1,7,22,29^ Graziani et al^12^ reported that only patients who lacked a history of periodontal surgery were included. Therefore, another limitation is the fact that the data would be more precise if all patients were treated with EMD in the same phase of periodontal therapy.

## Conclusion

In summary, the present systematic review with meta-analysis revealed that the use of EMD in combination with MIST improved both recession coverage and bone fill, whereas no difference in CAL or PD was observed. No statistically significant advantage was found for adjunctive EMD in a flapless approach in PPD or CAL gain, although reports of better BOP as well as treatment of periodontal pockets >5 mm was noted. Future research is needed to substantiate these findings, develop clinical guidelines, and identify potential factors leading to the observed outcomes.

## Figures and Tables

**Supplemental Table 1:** Risk of bias of randomised clinical trials

	Author/Year						
Domain	Fickl et al (2009)	Ribeiro et al (2011)	Graziani et al (2019)	Graziani et al (2020)	Jentsch et al (2021)	Schallhorn et al (2021)	Aimetti et al (2017)
Bias arising from the randomisation process	Some concern	Some concern	Low risk	Low risk	Low risk	Low risk	Low risk
Bias due to deviations from intended interventions	Low risk	Low risk	Low risk	Low risk	Low risk	Low risk	Low risk
Bias due to missing outcome data	Low risk	Low risk	Low risk	Low risk	Low risk	Low risk	Low risk
Bias in measurement of the outcome	Low risk	Low risk	Low risk	Low risk	Low risk	Low risk	Low risk
Bias in selection of the reported result	Low risk	Low risk	Low risk	Low risk	Low risk	Low risk	Low risk
Overall risk of bias judgment	Some concern	Some concern	Low risk	Low risk	Low risk	Low risk	Low risk

## Clinical relevance:

Preliminary data favors the use of EMD utilising MIST when compared to a flapless approach. Additional studies utilising similar periodontal conditions are needed for a more accurate comparison.
